# Application of a Portable Multi-Analyte Biosensor for Organic Acid Determination in Silage

**DOI:** 10.3390/s18051470

**Published:** 2018-05-08

**Authors:** Johanna Pilas, Yasemen Yazici, Thorsten Selmer, Michael Keusgen, Michael J. Schöning

**Affiliations:** 1 Institute of Nano- and Biotechnologies (INB), FH Aachen, Heinrich-Mußmann-Straße 1, 52428 Jülich, Germany; pilas@fh-aachen.de (J.P.); yasemen.yazici@alumni.fh-aachen.de (Y.Y.); selmer@fh-aachen.de (T.S.); 2 Institute of Pharmaceutical Chemistry, Philipps-Universität Marburg, 35037 Marburg, Germany; michael.keusgen@staff.uni-marburg.de; 3 Institute of Complex Systems 8 (ICS-8); Forschungszentrum Jülich GmbH, Wilhelm-Johnen-Straße 1, 52425 Jülich, Germany

**Keywords:** biosensor, ethanol, formate, d-/l-lactate, multi-analyte, silage

## Abstract

Multi-analyte biosensors may offer the opportunity to perform cost-effective and rapid analysis with reduced sample volume, as compared to electrochemical biosensing of each analyte individually. This work describes the development of an enzyme-based biosensor system for multi-parametric determination of four different organic acids. The biosensor array comprises five working electrodes for simultaneous sensing of ethanol, formate, d-lactate, and l-lactate, and an integrated counter electrode. Storage stability of the biosensor was evaluated under different conditions (stored at +4 °C in buffer solution and dry at −21 °C, +4 °C, and room temperature) over a period of 140 days. After repeated and regular application, the individual sensing electrodes exhibited the best stability when stored at −21 °C. Furthermore, measurements in silage samples (maize and sugarcane silage) were conducted with the portable biosensor system. Comparison with a conventional photometric technique demonstrated successful employment for rapid monitoring of complex media.

## 1. Introduction

Ethanol, formate, and lactate, existing in two isoforms (d- and l-lactate) are biological components with remarkable relevance in the clinical, food, environmental, and bioprocess industries. For medical surveillance, the levels of l-lactate and ethanol in human fluids (blood, serum, saliva, sweat, etc.) are of great interest, particularly in sports medicine [1] and for the evaluation of pathological conditions [2,3]. In mammalian cells, predominantly l-lactate is present, whereas d-lactate is mainly produced by microorganisms as an important intermediate or end-product in various metabolic pathways. Since ethanol as well as d- and l-lactate play a significant role in the alcohol, lactic, and malolactic fermentation processes, they serve as a valuable indicator for monitoring the freshness and quality of food products and beverages [4,5]. Formate is another relevant metabolite in many aerobic and anaerobic bacteria, with central significance in the methanogenic cascade [6,7]. In order to enable efficient operation of anaerobic digestors, feedstock with constant quality and composition are required. In this regard, silage preparation is an established method for preservation and storage of organic material. In anaerobic environments, different fermentation pathways occur, which are dominated by lactic acid-producing bacteria [8]. For successful control of ensiling or other fermentation processes, therefore, continuous monitoring of different intermediates (like ethanol, formate, d-lactate, and l-lactate) is mandatory. The most common analytical techniques for quantification of these analytes are high performance liquid chromatography [9,10], gas chromatography [11], and UV-VIS spectrophotometry. These methods generally possess high accuracy and sensitivity, but also may be associated with some potential drawbacks. For example, in some cases the expensive and advanced instruments require laborious sample pretreatment procedures for elimination of interfering substances and particles. These characteristics result in time-consuming and delayed analyses that are rather unsuited for real-time and on-site monitoring. In this regard, electrochemical biosensor arrays may provide an attractive and competitive alternative due to their selectivity and potential for miniaturization. Integration of several analyte-specific electrodes within an array enables construction of portable hand-held devices for monitoring purposes [12,13]. Although multitudinous electrochemical enzyme-based biosensors are described for detection of ethanol [14,15], formate [16,17], d-lactate, and l-lactate [18,19] individually, so far only few efforts have been described for combined analysis of these or other substrates [5,20,21].

We have recently reported on the development and extensive optimization of different enzyme-based biosensor arrays for the parallel determination of several analytes [22,23]. The combination of different dehydrogenases and a diaphorase, required for the amperometric detection principle and regeneration of the cofactor nicotinamide adenine dinucleotide (NAD) [24,25], allowed construction of electrochemical multi-parametric biosensors. The optimal working conditions and immobilization parameters were identified, resulting in improved sensor performance. There are several examples of economically successful biosensors [26], that are mostly designed as single-use and disposable devices, such as sensors for monitoring blood glucose levels of diabetic patients. From an operational point of view, however, reusable applicability is preferable, especially when costly biological materials are used as sensing elements. The stability of enzyme-based biosensors mainly depends on the activity of the employed proteins. For this reason, lot of research has been devoted to the immobilization procedure in order to extend the long-term applications [27,28].

The main objective of this work was to evaluate the capability of a multi-analyte biosensor for multiple usage in complex media from fermentation processes. For this reason, the storage stability under different conditions was investigated. A potential application as a portable sensor system for simultaneous determination of ethanol, formate, d-lactate, and l-lactate was demonstrated in pretreated samples of silage.

## 2. Material and Methods

### 2.1. Chemicals and Reagents

The enzymes alcohol dehydrogenase (ADH, Enzyme Commission number (EC) 1.1.1.1, 310 U mg^−1^) from *Saccharomyces cerevisiae*, formate dehydrogenase (FDH, EC 1.2.1.2, 0.49 U mg^−1^) from *Candida boidinii*, l-lactate dehydrogenase (l-LDH, EC 1.1.1.27, 174.5 U mg^−1^) from *Bacillus stearothermophilus*, d-lactate dehydrogenase (d-LDH, EC 1.1.1.28, 213 U mg^−1^) from *Lactobacillus leichmanii*, and diaphorase (DIA, EC 1.8.1.4, 51 U mg^−1^) from *Clostridium kluyveri* were purchased from Sigma-Aldrich (St. Louis, MO, USA). Bovine serum albumin (BSA), glutaraldehyde solution (GA) (25% in H_2_O), glycerol, potassium ferricyanide (K_3_[Fe(CN)_6_]), sodium d-lactate, and ethanol standard solution were also supplied by Sigma-Aldrich. Sodium formate, sodium l-lactate, and the cofactor nicotinamide adenine dinucleotide (NAD^+^) were obtained from AppliChem (Darmstadt, Germany). Potassium phosphate buffer (K_2_HPO_4_, KH_2_PO_4_) and H_2_SO_4_ were from Carl Roth GmbH & Co. KG (Karlsruhe, Germany).

All reagents were of analytical grade and were prepared in deionized water. Enzymatic stock solutions (ADH, DIA, FDH, d-LDH and l-LDH, respectively) were prepared in 0.1 mol L^−1^ potassium phosphate buffer (pH 7.5). The DIA solution was supplemented with 0.5 mmol L^−1^ flavin adenine dinucleotide.

### 2.2. Sensor Fabrication and Design

**Figure 1:**
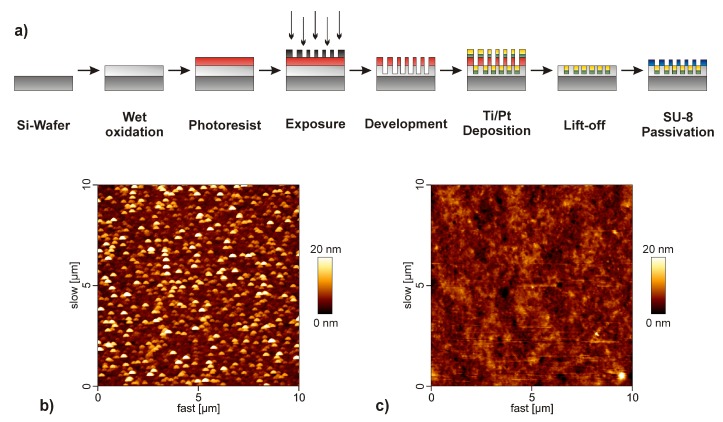
(**a**) Schematic process flow for fabrication of the silicon-based multi-analyte biosensor chip. Atomic force microscopy images (10 × 10 μm^2^) of (**b**) the blank platinum electrode and (**c**) the sensor surface after immobilization of d-lactate dehydrogenase (d-LDH) and diaphorase (DIA).

**Figure 2:**
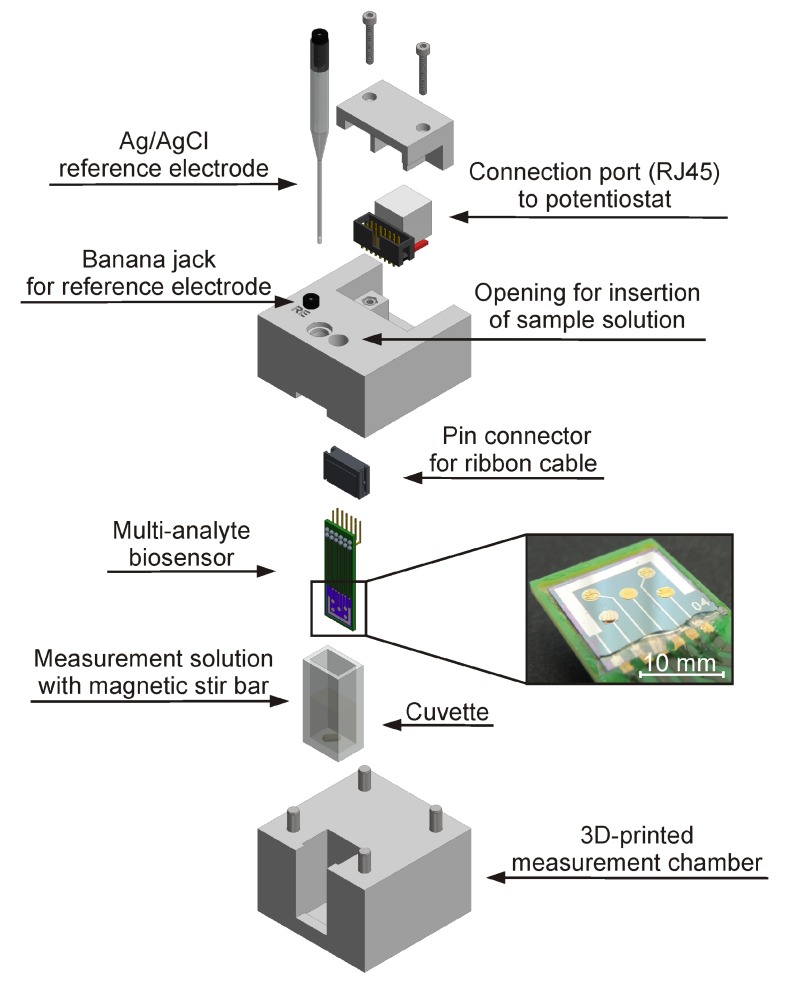
Explosion drawing of the compact three-dimensional (3D)-printed measurement set-up (60 × 60 × 70 mm^3^) for facile application of the multi-analyte biosensor. Close-up shows biosensor chip (14 × 14 mm^2^) with five working electrodes and an integrated counter electrode, incorporated into a printed circuit board, with immobilized enzyme membranes on the working electrodes.

For simultaneous amperometric detection of several substrates, the multi-analyte biosensor chip (14 × 14 mm^2^) features five circular working electrodes (each ⌀ 2 mm) and a rectangular counter electrode (40.5 mm^2^). The schematic steps for thin-film fabrication of the biosensor are depicted in Figure 1a [21]. Firstly, a 500-nm-thick layer of SiO_2_ was grown onto a p-type silicon wafer (⌀ 3 inch) by thermal wet oxidation at 1000 °C for 30 min. Deposition of photoresist AZ5214E (MicroChemicals GmbH, Ulm, Germany) was achieved by spin-coating at 4000 rpm for 30 s. A photolithographic step was used for patterning of the sensor layout by application of a custom-made mask and exposure of the photoresist film for 7.5 s at 8 mW cm^−2^. The photoresist film was then developed using developer AZ 326 MIF (micro resist technology GmbH, Berlin, Germany). Afterwards, electron beam evaporation was used for the deposition of 20 nm of titanium (Ti) as an adhesion layer and a 200-nm-thick layer of platinum (Pt). The Pt/Ti electrodes were patterned through a lift-off process in dimethyl sulfoxide (DMSO) using ultrasonication. For passivation an epoxy-based photoresist (SU-8 25, micro resist technology GmbH, Berlin, Germany) was spin-coated onto the wafer for 30 s at 1500 rpm, resulting in a 20-μm-thick layer [29,30]. A soft-bake process was used (9 min at 95 °C) for evaporation of the solvent. Initial cross-linking of the photoresist was then realized by exposure for 25 s at a wavelength of 356 nm. Following, a post-exposure bake step was performed for 4 min at 95 °C. The counter electrode, working electrodes, and contact pads were re-opened by development of the photoresist with the developer mr-Dev 600 (micro resist technology GmbH, Berlin, Germany). Finally, the wafer was diced into 16 single chips. Each chip was then cleaned and glued onto printed circuit boards and an electrical connection was established by ultrasonic wedge bonding with an AlSi bonding wire (Heraeus, Hanau, Germany). As presented in Figure 1b,c, the sensor morphology was characterized by atomic force microscopy (BioMat Workstation, JPK Instruments, Berlin, Germany) in tapping mode. The roughness of the surface after enzyme immobilization was comparable to that of the blank platinum electrode (4.4 nm). The close-up view in Figure 2 shows an image of the completed biosensor.

### 2.3. Sensor Preparation and Measurement Set-up

The multi-analyte biosensor was constructed by modification of each working electrode with a different analyte-specific dehydrogenase. Prior the immobilization process, the platinum surface was cleaned electrochemically in 0.5 M H_2_SO_4_ [22,31]. The enzymes were immobilized by chemical cross-linking with glutaraldehyde. This procedure was selected due to its simplicity and flexibility, which enables facile and low-cost immobilization of the different enzymes required for the biosensor array. Detailed description of the individual enzyme loading on each working electrode can be found elsewhere [22]. Briefly, for each working electrode an individual mixture was prepared, consisting of BSA, DIA, and the particular dehydrogenase (ADH, FDH, d-LDH, and l-LDH, respectively). Each solution was then carefully mixed with 2.4 vol % glutaraldehyde (with 10 vol % glycerol) and a volume of 1.5 μL was applied on the working electrode. One working electrode was functionalized only with the inert protein BSA, serving as a reference without catalytic activity. Passivation of the sensor chip with SU-8 promoted homogenous distribution of the enzymatic solutions within the entire area of the working electrode. After drying overnight in the fridge, the enzyme membranes were stable and ready to use.

Electrochemical measurements were performed in a conventional three-electrode set-up with a three-dimensional (3D)-printed chip holder made of composite material (ZP 151, 3D Systems GmbH, Darmstadt, Germany). Figure 2 shows an exploded view of the custom-made measurement cell, which provides facile electrical connection of the biosensor and miniaturized Ag/AgCl reference electrode (Sensolytics GmbH, Bochum, Germany). Simultaneous measurement of five electrodes was realized by application of a compact potentiostat EmStat3 with an integrated 16-channel multiplexer MUX16 (PalmSens BV, Houten, The Netherlands). Thereby, the working electrodes are constantly polarized by sharing a common reference and on-chip integrated counter electrode. The applied working potential was set to +0.3 V vs. the Ag/AgCl reference electrode for anodic oxidation of enzymatically produced K_4_[Fe(CN)_6_]. Sensor calibration and sample analysis were performed at room temperature in 2 mL of measurement solution (0.1 mol L^−1^ potassium phosphate buffer, pH 7.5, with 2.5 mmol L^−1^ NAD^+^ and 2.0 mmol L^−1^ K_3_[Fe(CN)_6_]). For a fast and homogenous distribution of the sample solution, a magnetic stir bar was used. These conditions represent the optimal working environment for the multi-analyte biosensor [22]. In comparison to a previously developed biosensor chip [22], the novel sensor design with the integrated counter electrode facilitates the miniaturization of the measurement set-up, resulting in reduced sample volume.

### 2.4. Sample Preparation and Analysis

Samples of maize and sugarcane silage were used for demonstration of applicability of the biosensor. Since liquid and clear sample solutions are required, especially for the reference measurement (photometric analysis), a pretreatment according to Carrez clarification was performed [24,32]. For this reason, 10 g (wet weight) of feedstock were incubated in 100 mL of deionized water for 30 min. Then, 15 mL of this solution were mixed with 3 mL Carrez I (0.68 mol L^−1^ K_4_[Fe(CN)_6_]·3H_2_O) and 3 mL Carrez II (2 mol L^−1^ ZnCl_2_). After incubation for 5 min, the solution was neutralized by addition of 7.5 mL NaOH (0.4 mol L^−1^) and 1.5 mL H_2_O. This mixture was centrifuged for 20 min at 7500 rpm and the particle-free supernatant was used for further analysis.

Prior to sample analysis, a calibration of the biosensor was performed by successive addition of a multi-analyte stock solution (40 mmol L^−1^ of each analyte) and recording of the corresponding increase in current signal. The biosensor was then washed with potassium phosphate buffer (0.1 mol L^−1^, pH 7.5) and used for determination of organic acids in real samples. Defined volumes of sample solution were added to the measurement solution in order to generate different dilutions. Based on the obtained calibration curves and the dilution factor of the sample, the concentration of each analyte was calculated (defined as weight of analyte per wet weight of silage). Reference analytics were performed with commercial photometric kits following the manufacturer’s instructions (Megazyme International, Wicklow, Ireland).

## 3. Results and Discussion

### 3.1. Simultaneous Measurement Procedure

**Figure 3:**
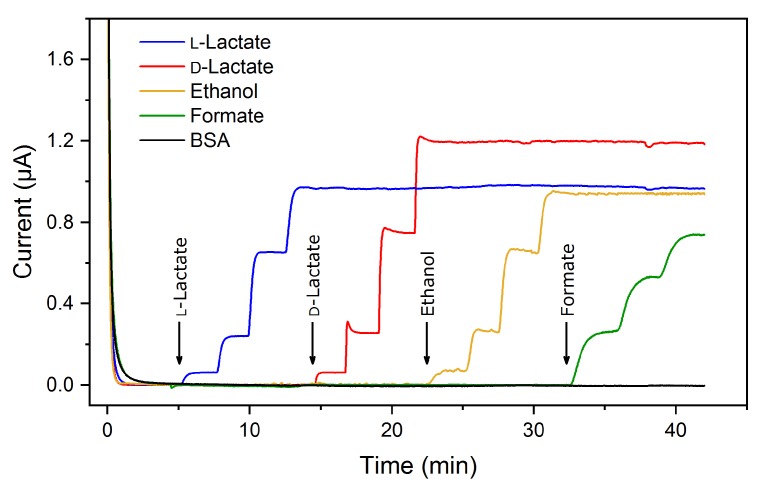
Chronoamperometric current responses of the multi-analyte biosensor to successive addition of single analyte stock solutions (l-lactate, d-lactate, ethanol, and formate) in 100 mM potassium phosphate buffer (pH 7.5). A working electrode with immobilized bovine serum albumin (BSA) served as a blank signal.

Integration of several analyte-specific working electrodes that rely on the same detection principle within one biosensor chip facilitates the construction of multi-analyte systems. In this work, different NAD^+^-dependent dehydrogenases were used for simultaneous determination of ethanol, formate, d-lactate, and l-lactate at the same applied working potential. Figure 3 shows the method of simultaneous operation for detection of different analytes. Multi-analyte assays in particular depend on appropriate substrate specificity in order to exclude potential interferences. The cross-talk-free performance of the developed biosensor is demonstrated by successive addition of each analyte separately. It becomes obvious that only the corresponding electrode reacts with an increase in the current signal when the analyte is present in the measurement solution. Furthermore, the blank signal (BSA electrode) remains constant throughout the measurement. This sensor characteristic allows the simultaneous quantification of several analytes within a shorter analysis time compared to the single detection of each substrate.

### 3.2. Evaluation of Storage Stability

**Figure 4:**
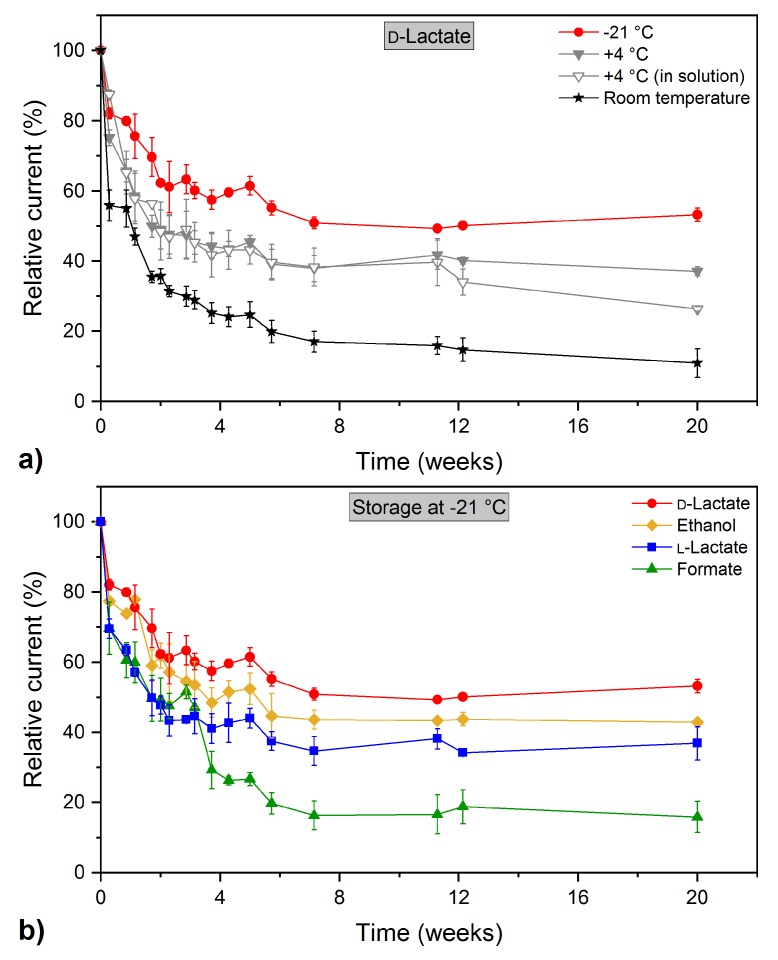
(**a**) Relative current signal as a function of storage time of the d-lactate-sensing electrode in the presence of 1 mmol L^−1^ d-lactate in different storage conditions (−21 °C, +4 °C, +4 °C in buffer solution and room temperature, respectively); (**b**) Storage stability of the multi-analyte biosensor stored in a freezer at −21 °C for a period of 20 weeks (*n* = 3 sensors) .

The storage stability of any biosensor is an important aspect when it comes to the requirement of long-term applications [27]. The decisive factor in this context is the immobilization of the biological component with ideally minimum enzyme leakage and denaturation. Stability of the multi-analyte biosensor was examined by storage under dry conditions at −21 °C, +4 °C, and room temperature, as well as immersion in buffer solution at +4 °C (0.1 mol L^−1^ potassium phosphate buffer, pH 7.5). The response to 1 mmol L^−1^ of each analyte (ethanol, formate, d-lactate, and l-lactate, respectively) was investigated regularly for a period of 20 weeks. After each measurement the sensors were washed with phosphate buffer (0.1 mol L^−1^, pH 7.5) and stored under the particular conditions until further use. In Figure 4a the results are exemplarily presented for the d-lactate-sensing electrode. During the first four weeks of storage, there was a sharp decline in the sensor response for all storing conditions tested. Subsequently, the signal decreased gradually and between weeks 7 and 20 the obtained current remained almost stable. The d-lactate electrode retained 53% of its initial signal after more than 4 months of intermittent application when stored at −21 °C. Storage in the fridge at +4 °C resulted in 37% of original response (dry state) and 26% (in solution), respectively. At ambient temperatures the d-lactate electrode exhibited the greatest decrease in sensor response (11%), indicating that storing the sensor under this condition is not suitable for long-term applications.

The storage stability at −21 °C of the individual electrodes of the multi-analyte biosensor is depicted in Figure 4b. Within the first 7 weeks the relative response of the different electrodes declined rapidly. Thereafter, the overall sensor performance did not change significantly. The biosensor showed good stability and maintained 43% and 37% of its initial response after 140 days for the ethanol and l-lactate electrode, respectively. The formate electrode was the least stable, characterized with 16% of original maximum obtained current at the end of investigation. Despite the repeated freezing and thawing, which is known to be harmful for enzyme stability [33,34], storage at −21 °C proved to be best for preservation of the sensor stability. The l-lactate electrode was the only one which showed improved stability after storage at 4 °C in buffer solution (see Table 1). These findings may be associated with the applied l-LDH from *B. stearothermophilus*, which is probably less stable at freezing temperatures. An overview of different enzyme-based biosensors and the characteristics of their storage stability is summarized in Table 1. Hereby, stability is defined as storage time *t_L50_* necessary for the initial sensor response to decrease by 50% [35]. In most of these studies the biosensors were stored at +4 °C in buffer solution and exhibited under this condition similar stability over several days or weeks in comparison to the multi-analyte biosensor stored at −21 °C. The stability of enzyme-based biosensors is particularly influenced by the applied procedure for immobilization of the biological component. More gentle immobilization treatments than cross-linking with glutaraldehyde can facilitate long-term storage for up to 5 months at room temperature [36].

Although the individual electrodes of the multi-analyte biosensor showed different characteristics in their storage stability, the overall capability of long-term and multiple usage was demonstrated successfully. For practical applications, however, a recalibration of the biosensor is mandatory due to steady decrease in sensor response.

### 3.3. Measurement of Organic Acids in Silage

**Figure 5:**
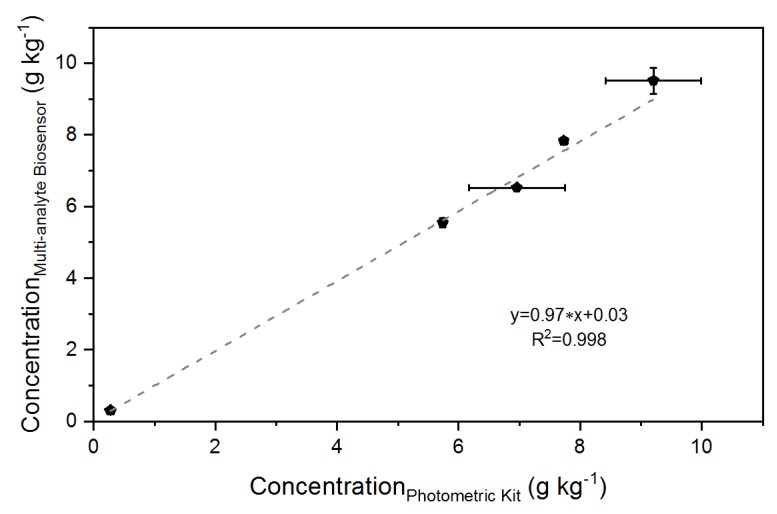
Comparison between the organic acid concentrations (ethanol, formate, d-lactate, and l-lactate) obtained by the amperometric and photometric method.

The developed multi-analyte biosensor system was used for determination of the organic acid concentration in two different silage samples, namely maize and sugarcane. These energy crops are typical feedstocks used for biogas production from renewable resources [42,43]. Since the methane yield during the fermentation process is mainly influenced by the composition of the applied substrate, systematic and regular characterization of the silage is of huge importance for an efficient conversion of organic material to biogas [44,45,46]. The amount of organic acids was also measured with a commercial reference technique. This method likewise is based on an enzymatic detection principle (spectrophotometric analysis of NADH increase at a wavelength of 340 nm). However, in this case each analyte has to be quantified separately, resulting in a more time-consuming and laborious analysis procedure. Table 2 and Figure 5 illustrate the concentrations obtained by both analytical methods. The results demonstrate a good correlation (R^2^ = 0.998) between the two techniques for all four analytes with an apparent recovery in the range of 93.7 to 109.3% [47]. Although sometimes values greater than 100% were obtained, overall the multi-analyte biosensor provides the advantage of facile and simultaneous determination of several analytes in complex media in comparison to the photometric analysis. The particular sensor characteristics of each electrode of the multi-analyte array have been extensively optimized in earlier studies [22]. Detection of formate and d-lactate was performed with a sensitivity of 20.5 and 28.4 μA mmol^−1^ L cm^−2^, respectively. The ethanol and l-lactate electrodes were characterized by slightly higher sensitivities (35.7 and 37.2 μA mmol^−1^ L cm^−2^, respectively). Based on a signal-to-noise ratio of 3, the limit of detection (LOD) for ethanol, l-lactate, and d-lactate was 0.7, 0.7, and 0.9 μmol L^−1^, respectively. The obtained values for the LOD for these three electrodes (ethanol, d-lactate, and l-lactate) were in a similar or better range in comparison to other enzyme-based biosensors reported for the detection of ethanol [14], d-lactate, and l-lactate [19]. These sensor characteristics enabled successful quantification of several organic acids in silage samples. However, no formate was detectable in the sugarcane silage, probably because the concentration of this acid was below the LOD of the corresponding formate electrode (LOD = 1.3 μmol L^−1^). In literature, various enzyme-based formate biosensors are described, which utilize a different detection principle. Electrocatalytic oxidation of NADH on a glassy carbon electrode, modified with 3,4-dihydroxybenzaldehyde and FDH, allowed formate sensing with a LOD of 50 μmol L^−1^ [16]. A detection limit of 0.198 μmol L^−1^ was realized by combination of FDH with a salicylate hydroxylase for monitoring the enzymatic oxygen consumption with a Clark electrode [17]. When it comes to the development of biosensors, consideration of the intended field of application and required sensor performances is also of great interest. For the analysis of the acid composition in silage samples, typically concentrations below the micromolar range are not of great importance, because they will not influence the biogas fermentation process. In this regard, the presented characteristics of the developed multi-analyte biosensor system meet the requirements for the desired application, and compared to single biosensors for individual analyte detection, the array configuration permits a faster analysis.

## 4. Conclusions

Within this study, a multi-analyte biosensor system for the detection of four different organic acids is introduced. Simultaneous and cross-talk-free measurements of ethanol, formate, d-lactate, and l-lactate were realized by immobilization of analyte-specific enzymes on each working electrode. The storage stability of the presented biosensor array was investigated at different storage conditions for a period of 20 weeks. For this reason, sensors were stored in buffer solution at +4 °C and dry at −21 °C, +4 °C, and room temperature, respectively. Storing at −21 °C proved to be the best storage option, although repeated freezing and thawing affected each sensor element differently. With almost 53% of the initial activity after 140 days of intermittent usage, the d-lactate electrode exhibited the highest long-term stability. The formate electrode retained only 16% of its initial sensor signal.

Successful application of the biosensor for quantification of organic acids in complex media was demonstrated in silage samples (maize and sugarcane). Data obtained by amperometric measurements were in good agreement with a commercial reference technique. In comparison to conventional analytical methods, the described biosensor system offers the advantages of rapid and simultaneous detection, and capability for repeated application due to long-term stability. For particular requirements, the flexible system can be easily enhanced with additional electrodes for further analytes [23]. Future investigations will focus on application of the biosensor in different biological samples and evaluation of the compact and portable measurement device for long-term and on-site monitoring of fermentation processes. In this regard, the construction of a reagentless biosensor is planned through the coimmobilization of the cofactors NAD^+^ and K_3_[Fe(CN)_6_] for improved ease of use.

## Figures and Tables

**Table 1:** Comparison of the storage stability of various enzyme-based biosensors (d: day; m: month; SHL: salicylate hydroxylase; POx: pyruvate oxidase; PCS: poly(carbamoyl)sulfonate; PVA: polyvinyl alcohol; MWCNT: multiwalled carbon nanotube).

Analyte	Enzymes	Detection	Immobilization	Stability *t_L_*_50_	Storage	Reference
d-Lactate	d-LDH	Toluidine blue O	Carbon paste	<30 d	4 °C	[37]
d-Lactate	d-LDH+DIA	Fe[CN)_6_]^−4^	Entrapment	40 d	4 °C in buffer	[25]
d-Lactate	d-LDH+DIA	Fe[CN)_6_]^−4^	Glutaraldehyde	50 d	−21 °C	Present work
l-Lactate	l-LDH+SHL+POx	O_2_ consumption	PCS Hydrogel	11 d	4 °C in buffer	[38]
l-Lactate	l-LDH+DIA	Fe[CN)_6_]^−4^	Graphite powder	>5 m	RT, sealed	[36]
l-Lactate	l-LDH+DIA	Fe[CN)_6_]^−4^	Glutaraldehyde	40 d	4 °C in buffer	Present work
l-Lactate	l-LDH+DIA	Fe[CN)_6_]^−4^	Glutaraldehyde	14 d	−21 °C	Present work
Ethanol	ADH	Toluidine blue O	Glutaraldehyde	20 d	4 °C in buffer	[39]
Ethanol	ADH	NADH	Glutaraldehyde	35 d	−20 °C	[40]
Ethanol	ADH	NADH	PVA–MWCNT	7 d	4 °C in buffer	[41]
Ethanol	ADH+DIA	Fe[CN)_6_]^−4^	Glutaraldehyde	22 d	−21 °C	Present work
Formate	FDH+SHL	O_2_ consumption	PVA matrix	10 d	23 °C	[17]
Formate	FDH+DIA	Fe[CN)_6_]^−4^	Glutaraldehyde	20 d	−21 °C	Present work

**Table 2:** Determination of organic acids in silage samples using two different analytical techniques (BD: below the lower detection limit). The apparent recovery was defined as the observed value/reference value.

Sample	Analyte	Photometric Kit (g kg^−1^)	Multi-analyte Biosensor (g kg^−1^)	Apparent Recovery (%)
Maize Silage	d-Lactate	7.73 ± 0.06	7.83 ± 0.07	101.3
	l-Lactate	5.74 ± 0.03	5.54 ± 0.13	96.5
	Ethanol	6.96 ± 0.79	6.52 ± 0.06	93.7
	Formate	0.27 ± 0.03	0.30 ± 0.04	109.3
Sugarcane Silage	d-Lactate	0.29 ± 0.05	0.30 ± 0.03	103.5
	l-Lactate	0.28 ± 0.01	0.25 ± 0.07	106.2
	Ethanol	9.20 ± 0.78	9.51 ± 0.36	103.3
	Formate	BD	BD	-

## Abbreviations

The following abbreviations are used in this manuscript:

ADH	Alcohol dehydrogenase
BD	Below lower detection limit
BSA	Bovine serum albumin
d	Day
DIA	Diaphorase
FDH	Formate dehydrogenase
d-LDH	d-Lactate dehydrogenase
l-LDH	l-Lactate dehydrogenase
LOD	Limit of detection
m	Month
MWCNT	Multiwalled carbon nanotube
POx	Pyruvate oxidase
PCS	Poly(carbamoyl)sulfonate
PVA	Polyvinyl alcohol
SHL	Salicylate hydroxylase
*t_L_*_50_	Storage time necessary for initial sensor signal to decrease by a factor of 50%
